# Uncovering and Predicting the Dynamic Process of Collective Attention with Survival Theory

**DOI:** 10.1038/s41598-017-02826-6

**Published:** 2017-06-01

**Authors:** Peng Bao, Xiaoxia Zhang

**Affiliations:** 1 School of Software Engineering, Beijing Jiaotong University, Beijing, China; 2 School of Economics and Management, Tsinghua University, Beijing, China

## Abstract

The subject of collective attention is in the center of this era of information explosion. It is thus of great interest to understand the fundamental mechanism underlying attention in large populations within a complex evolving system. Moreover, an ability to predict the dynamic process of collective attention for individual items has important implications in an array of areas. In this report, we propose a generative probabilistic model using a self-excited Hawkes process with survival theory to model and predict the process through which individual items gain their attentions. This model explicitly captures three key ingredients: the intrinsic attractiveness of an item, characterizing its inherent competitiveness against other items; a reinforcement mechanism based on sum of each previous attention triggers; and a power-law temporal relaxation function, corresponding to the aging in the ability to attract new attentions. Experiments on two population-scale datasets demonstrate that this model consistently outperforms the state-of-the-art methods.

## Introduction

The subject of collective attention is central to an information era from knowledge database to online media, where millions of people are inundated with the explosive growth of user generated items^1–3^. In the heart of collective attention lies a competing process through which a few items become popular while most fade with time^4–7^. For example, papers increase their visibility by competing for citations from new papers^8–10^, tweets or Hashtags in Twitter become more popular as being re-tweeted,^11, 12^, videos on YouTube or stories on Digg gain their popularity by striving for views or votes^13, 14^. Therefore, to understand the process underlying attention in large groups and predict the dynamic process of collection attention for individual items within a dynamically evolving system not only probes our understanding of complex systems, but also has important implications in a wide range of domains, including viral marketing, traffic control, public opinion monitoring, etc.^15–18^. However, to predict the dynamics of collective attention is challenging since numerous factors can affect the attention gathered by online content. Moreover, attention is very asymmetric and broadly-distributed^19, 20^. Early studies devote to characterizing the distribution of the collective attention over an aggregation of user generated items^21–23^ and making prediction on the final scale of attentions by exploiting temporal correlations^24–26^.

In recent year, there has been heightened research interest regarding the predictive modeling of the dynamics of collective attention for online content^27, 28^. In general, current models fall into two main paradigms, each with known strengths and limitations. One focuses on making predictions by exploring relevant factors and applying standard regression or classification methods^29–31^. These models reveal many effective factors for prediction. However, there are still numerous factors to be investigated and they lack predictive power for the dynamics of collective attention for individual items. The other line of enquiry, in contrast, treats the dynamics as time series, making predictions by fitting these time series into certain class of functions^32, 33^. Despite their initial success in certain domains, these models are deterministic and ignore the underlying arrival process of attentions. Recently, more sophisticated models have been proposed to simulate the dynamics of attentions for individual items, treating the diffusion process as a reinforced Poisson process^34, 35^ or a double stochastic process^36^. However, these models usually assume an aggregate stochastic process without distinguishing the triggering effects of different attentions in the diffusion-and-reaction process. Therefore, we still lack an effective method to uncover and predict the dynamics of collective attention.

In this report, we propose a generative probabilistic model using a self-excited Hawkes process with survival theory to model and predict the dynamic process through which individual items gain their attentions. This model explicitly captures three key ingredients simultaneously: the intrinsic attractiveness of an item, characterizing its inherent competitiveness against other items; a reinforcement mechanism based on sum of each previous attention triggers, documenting the well-known “rich-get-richer” phenomenon; and a power-law temporal relaxation function, corresponding to the aging in the ability to attract new attentions. We validate the proposed model by applying it on two different types of population-scale datasets, of which one is a citation dataset, the other one a micro-blogging dataset. Experimental results demonstrate that our proposed model consistently outperforms the state-of-the-art methods on two datasets.

## Material and Methods

### Data description

We use two population-scale datasets for this study, as follows.

- *APS*: It comprises the papers published in all the journals in American Physical Society from 1893 to 2009, consisting of 245,365 authors, 463,344 papers, and 4,692,026 citations. For each paper, the dataset includes title, DOI, PACS code, date of publication (day, month, year), names and affiliations of every author, a list of the previous papers cited, and so on (http://journals.aps.org/datasets).
- *WEIBO*: It is collected from the most popular micro-blogging service in China, namely Sina Weibo, which has more than 300 million registered users and generates about 100 million messages per day. Here we only use the messages that were originally posted between July 1, 2011 and July 31, 2011. There are 2.6 million messages. For each message, we collect its forwardings between July 1, 2011 and August 31, 2011 (http://www.wise2012.cs.ucy.ac.cy/challenge.html).

See Supplementary Section S1 for details.

### The model

We now introduce the proposed generative probabilistic model from the perspective of individual items. Supposing that there are a set of time moments {*t*_*i*_} (1 ≤ *i* ≤ *N*) which denote the occurrence time of each attention for individual item *d* during observed time period [0,*T*]. Here, *N* is the total number of attentions. Without loss of generality, we have 0 = *t*_0_ ≤ *t*_1_ ≤ *t*_2_ ≤ ... ≤ *t*_*i*_ ≤ ... ≤ *t*_*N*_ ≤ *T*. In this report, we model its dynamic process of attentions using a *self-excited Hawkes process* ^37^, incorporating three key ingredients simultaneously: (1) attractiveness of an item, characterizing its inherent competitiveness against other items; (2) a reinforcement mechanism based on sum of previous attention triggers, capturing the well-known “richer-get-richer” phenomenon; (3) a general temporal relaxation function corresponding to the aging effect, characterizing time-dependent attractiveness of individual items. Taken these three factors together, for an individual item *d*, we model its dynamics of attentions characterized by the rate function *λ*(*t*) as

1 $$\lambda (t)=\mu +\sum _{\mathrm{0 < }{t}_{i} < t}\phi (t-{t}_{i}),$$

where *μ* is the intrinsic attractiveness of the item, *φ*(*τ*) is the relaxation function that characterizes the temporal inhomogeneity due to the aging effect. The explicit form of *φ*(*τ*) will be investigated in the following section. Our model generalize the reinforcement function as the sum of each previous attention triggers with time decaying, instead of the total count of attentions^34, 35^.

The length of time interval between two consecutive attentions follows a self-excited Hawkes process. Therefore, given that the (*i* − 1)-th attention arrives at *t*_*i*−1_, the probability that the *i*-th attention arrives at *t*_*i*_ follows

2 $$p({t}_{i}|{t}_{i-1})={e}^{-{\int }_{{t}_{i-1}}^{{t}_{i}}\lambda (t)dt}\lambda ({t}_{i}),$$

which is the product of the survival and hazard functions. Specifically, the survival function ${e}^{-{\int }_{{t}_{i-1}}^{{t}_{i}}\lambda (t)dt}$ captures the probability that no attention arrives in the interval (*t*_*i*−1_,*t*_*i*_), and the hazard function *λ*(*t*_*i*_) captures the instantaneous rate of the *i*-th attention arrives a*t t*_*i*_. Similarly, because there is no attention arrives between *t*_*N*_ and *T*, the probability can be written as

3 $$p(T|{t}_{N})={e}^{-{\int }_{{t}_{N}}^{T}\lambda (t)dt}\mathrm{.}$$

Assuming that attentions during different time intervals are statistically independent, by incorporating equation (2) and (3), the likelihood of observing the dynamics {*t*_*i*_} during time interval [0,*T*] follows

4 $$L=p(T|{t}_{N})\prod _{i=1}^{N}p({t}_{i}|{t}_{i-1})\mathrm{.}$$

**Figure 1:**
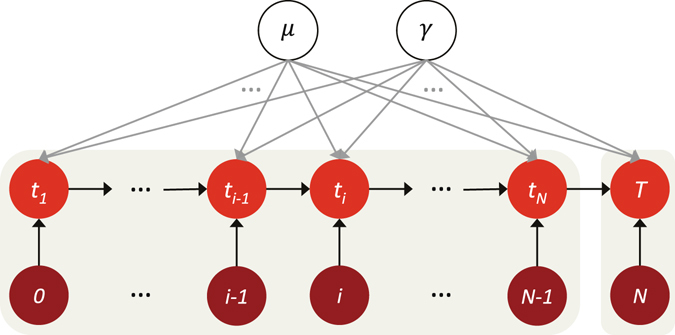
Graphical representation of the generative probabilistic model. Here {*t*_*i*_} (1 ≤ *i* ≤ *N*) is a set of time moments which denote the occurrence time of each attention for an individual item during observed time period [0,*T*]. *μ* and *γ* represent the parameters in the model. This generative probabilistic model explicitly models the arrival process of attentions.

For clarity, we illustrate the proposed model in the graphical representation, as shown in Fig. 1.

## Results

### Empirical validation of power-law temporal relaxation function

**Figure 2:**
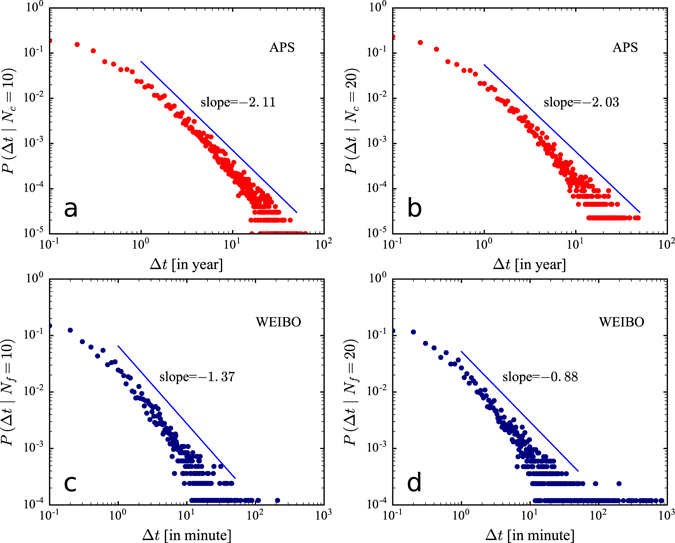
Empirical validation of temporal relaxation function. **(a)** *P*(Δ*t*) when the number of citations *N*_*c*_ change from 10 to 11 in the APS dataset. **(b)** *P*(Δ*t*) when the number of citations *N*_*c*_ change from 20 to 21 in the APS dataset. **(c)** *P*(Δ*t*) when the number of forwardings *N*_*f*_ change from 10 to 11 in the WEIBO dataset. **(d)** *P*(Δ*t*) when the number of forwardings *N*_*f*_ change from 20 to 21 in the WEIBO dataset. We find that the temporal relaxation function roughly follows a power-law distribution on two datasets. Note that a smaller power exponent indicates a slower decaying speed of the attractiveness of items.

The temporal relaxation function *φ*(*τ*) can be measured directly from the real data. As shown in the rate function in equation (1), the temporal dynamics of an item is controlled by three forces, which are difficult to separate from each other. Hence to determine the specific form of temporal relaxation function, we need to control the other factors, isolating the temporal decay. To achieve this we should group items with the same attractiveness and cumulative attentions, and look at the time when they receive next attention. However, we do not know the attractiveness beforehand. Therefore, by aggregating different items, we will measure a superposition of different temporal relaxation functions. We therefore select papers published between 1950 and 1970 in the APS dataset with fixed cumulative citations *N*_*c*_, and track the moment when their citations changed from *N*_*c*_ to *N*_*c*_ + 1. We denote Δ*t* the time interval between two consecutive attentions and measure Δ*t* in years, i.e. years passed when *N*_*c*_ → *N*_*c*_ + 1 took place. Here *P*(Δ*t*|*N*_*c*_) is the probability that a paper gets cited after time Δ*t* elapsed with fixed cumulative citations *N*_*c*_, capturing a paper’s attractiveness to the research community. In Fig. 2a and b, we present the distribution of *P*(Δ*t*|*N*_*c*_) for fixed *N*_*c*_ = 10 and *N*_*c*_ = 20. We find that *P*(Δ*t*|*N*_*c*_) roughly follows a power-law distribution with an exponent 2.11 for *N*_*c*_ = 10 and an exponent 2.03 for *N*_*c*_ = 20 respectively, indicating that collective attention is allocated in a rather asymmetric way, with a burst of rapidly arriving attentions followed by long periods of no attention.

In addition, it is similar to measuring *P*(Δ*t*) from empirical data in the WEIBO dataset. We roughly consider messages which are posted in a fixed time period and receive the same number of forwardings in one hour after being posted as having the same attractiveness. By selecting messages with same number of forwardings, the reinforcement is also controlled. Therefore we select messages posted between 10 am and 12 am with fixed cumulative number of forwardings *N*_*f*_ in the first hour after being posted, and track the moment when their number of forwardings changed from *N*_*f*_ to *N*_*f*_ + 1. Note that we measure Δ*t* in minutes to track the time interval due to the granularity of time scale^26, 34^. As shown in Fig. 2c and d, *P*(Δ*t*|*N*_*f*_) also displays a power-law distribution with an exponent 1.37 for *N*_*f*_ = 10 and an exponent 0.88 for *N*_*f*_ = 20 respectively.

The result reflects the emergence of bursty human behaviors^4^, exhibiting the temporal nature of collective attention. Meanwhile, although the dynamic behaviors on both datasets obey the power-law temporal scaling, the power exponents are quite different. Therefore, we need to assign an item-specific exponent to capture the inhomogeneous aging effect among individual items. Hence in this report, we model the aging effect by adopting a power-law temporal relaxation function for individual items as follows

5 $$\begin{array}{c}\phi (\tau )\propto {\tau }^{-\gamma }\mathrm{.}\end{array}$$

Note that *γ* is one of the parameters of our proposed model, which characterizes the item-specific aging effect and can be estimated by maximum likelihood estimation methods in the section below.

### Parameter estimation and prediction

By substituting the power-law temporal relaxation function in equation (5) into the general rate function in equation (1), we can get the specific form of rate function for dynamics {*t*_*k*_} as

6 $$\lambda (t)=\mu +\sum _{\mathrm{0 < }{t}_{i} < t}{(t-{t}_{i})}^{-\gamma }\mathrm{.}$$

Next, by substituting equation (6) into the likelihood function in equation (4) and taking logarithm, we can get the log-likelihood for the dynamics {*t*_*k*_} up to *T* as

7 $$\ell =ln(p(T|{t}_{N})\prod _{i\mathrm{=1}}^{N}p({t}_{i}|{t}_{i-1}))=\sum _{i\mathrm{=1}}^{N}\,\mathrm{ln}\,\lambda ({t}_{i})-\sum _{i=1}^{N}{\int }_{{t}_{i-1}}^{{t}_{i}}\lambda (t)dt-{\int }_{{t}_{N}}^{T}\lambda (t)dt=\frac{1}{1-\gamma }X-\mu T,$$

where $$X={\sum }_{i=1}^{N}(\mathrm{(1}-\gamma )\mathrm{ln}(\mu +{\sum }_{{t}_{j} < {t}_{i}}{({t}_{i}-{t}_{j})}^{-\gamma })-{(T-{t}_{i})}^{1-\gamma })$$

Then we utilize maximum likelihood estimation methods to estimate the parameters in the proposed model. For parameter {*μ*,*γ*}, the optimal values can be found by maximizing the log-likelihood in equation (7) using the gradient ascent method. See Supplementary Section S2 for details.

Here, we denote the optimal values for parameters {*μ*,*γ*} as {*μ* ^*^, *γ* ^*^}. With the obtained parameters, the model can be used to predict the expected number of attentions gathered by item *d* up to any given time *t*, which is denoted as *c*(*t*). Incorporating with the rate function in equation (6), for *t* > *T*, we treat the prediction task as the following differential equation

8 $$\frac{{\rm{d}}c(t)}{{\rm{d}}t}=\mu +\sum _{\mathrm{0 < }{t}_{i} < t}{(t-{t}_{i})}^{-\gamma }$$

with the boundary condition *c*(*T*) = *N*. Solving this differential equation, we obtain the prediction function

9 $$c(t)=N+{\mu }^{\ast }(t-T)+\sum _{\mathrm{0 < }{t}_{i} < t}\frac{1}{1-{\gamma }^{\ast }}({(t-{t}_{i})}^{1-{\gamma }^{\ast }}-{(T-{t}_{i})}^{1-{\gamma }^{\ast }})\mathrm{.}$$

### Experiment results

To compare the predictive power of our proposed model against other models, we introduce two widely-used models that have been used or can be used to model and predict the dynamics of collective attention: the WSB model^34^ and the SEISMIC model^36^. See Supplementary Section S3 for details.

In order to validate the prediction performance of all the prediction models, we utilize two evaluation metrics: Mean Absolute Percentage Error (*MAPE*) and *Accuracy*. Let *c*_*d*_(*t*) be the observed number of attentions for an item *d* up to time *t*, and ${\hat{c}}_{d}(t)$ be the predicted value.

- *MAPE* measures the average deviation between the predicted and empirical number of attentions over an aggregation of items. For a dataset of *D* items, the *MAPE* is defined as

$$MAPE=\frac{1}{D}\sum _{d=1}^{D}|\frac{{\hat{c}}_{d}(t)-{c}_{d}(t)}{{c}_{d}(t)}|\mathrm{.}$$

- *Accuracy* measures the fraction of items, correctly predicted under a given error tolerance *ε*. Specifically, the *Accuracy* of prediction over *D* items is defined as

$$Accuracy=\frac{1}{D}\sum _{d\mathrm{=1}}^{D}{\rm{I}}[|\frac{{\hat{c}}_{d}(t)-{c}_{d}(t)}{{c}_{d}(t)}|\le \varepsilon ]\mathrm{.}$$

where I[*X*] is an indicator function which return 1 if the statement *X* is true and 0 otherwise. In this report, the threshold *ε* is set as 0.1.

Therefore, for the APS dataset, we set the training period *T* as 10 years and then predict the number of citations for each paper from the 1st to 20th year after the training period. Similarly, for the WEIBO dataset, the training period is 6 hours and we predict the number of forwardings for each message from the 1st to 42nd hour after the training period.

**Figure 3:**
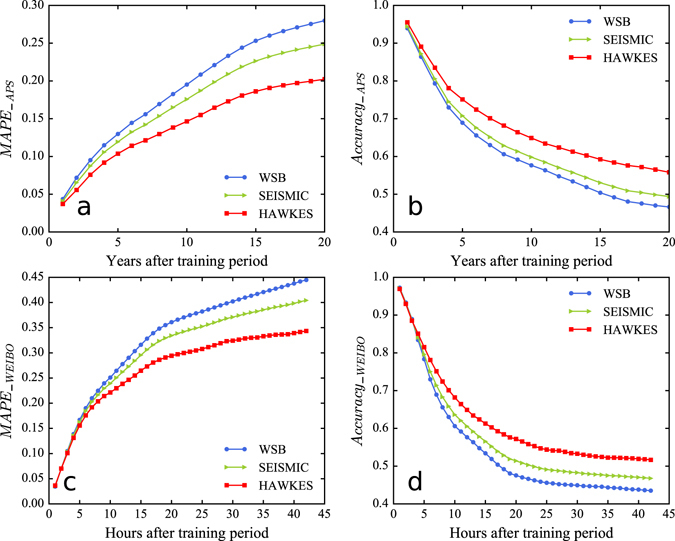
Prediction comparison for different models. **(a)** *MAPE* of model prediction with respect to the 1st to 20th year after the training period in the APS dataset. **(b)** *Accuracy* of model prediction with respect to the 1st to 20th year after the training period in the APS dataset. **(c)** *MAPE* of model prediction with respect to the 1st to 42nd hour after the training period in the WEIBO dataset. **(d)** *Accuracy* of model prediction with respect to the 1st to 42nd hour after the training period in the WEIBO dataset. We find that the proposed model (HAWKES) consistently outperforms the state-of-the-art methods (WSB and SEISMIC), exhibiting lower error (**a**,**c**) and higher accuracy (**b**,**d**).

Figure 3 shows the comparison results of these models with respect to different prediction time on two datasets. We find that the proposed model consistently outperforms the state-of-the-art methods, exhibiting lower error (Fig. 3a and c) and higher accuracy (Fig. 3b and d).

Furthermore, we carry out extensive experiments on two datasets to examine the prediction performance of different models when the training period varies. To be specific, we apply these models on the APS dataset with the training period varying from 2 to 14 years. And we fix the prediction time *t* to be 20 years after publication. For the WEIBO dataset, we change the training period from 1 to 8 hour. Since most messages in the dataset stop receiving more forwarding after being posted for 48 hours^25^, we fix the prediction time *t* to be 48 hours to check the ability for different models in predicting the final number of received attentions. We use MAPE to measure the prediction performance.

**Figure 4:**
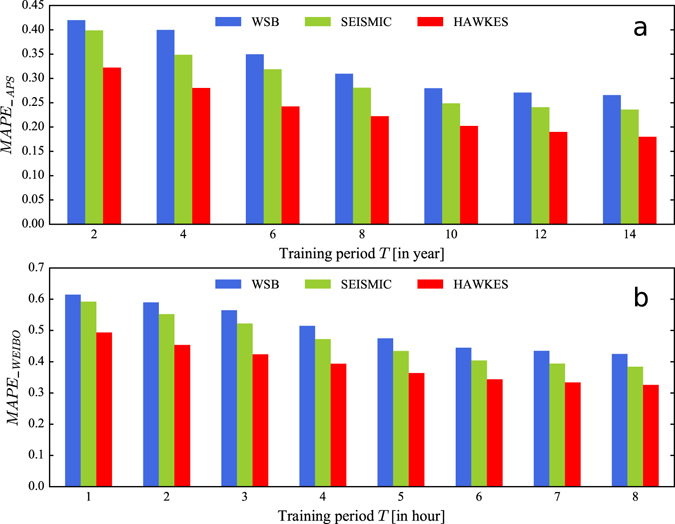
Prediction comparison for different models with the variation of training period. **(a)** *MAPE* of model prediction with respect to different length of training period in the APS dataset. **(b)** *MAPE* of model prediction with respect to different length of training period in the WEIBO dataset. We find that the proposed model (HAWKES) performs the best on the entire range of training period on two datasets. It also shows that the MAPE decreases as the training period increases.

Are shown in Fig. 4, for all models, the MAPE decreases as the training period increases on two datasets, indicating that increasing the training period can improve the prediction performance for all the models. More importantly, we find that the proposed model performs the best on the entire range of training period on two datasets, indicating the effectiveness of the proposed model. In addition, we can also see that the rate at which MAPE declines slows down quickly. This means the marginal gain for performance improvement diminishes with the increasing of the training period.

### Analysis of model parameters

In our model, there are in total two parameters {*μ*,*γ*} and they are derived from the model learning process. Here we investigate the characteristics of the learned parameters.

**Figure 5:**
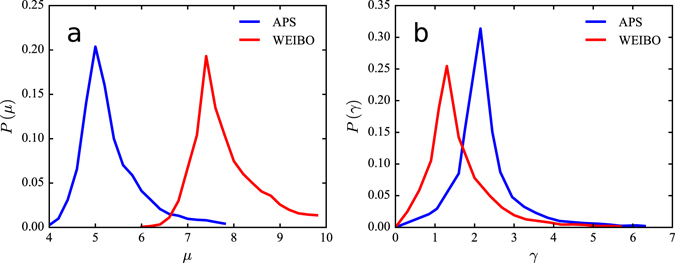
Distribution of model parameters in two datasets. **(a)** Distribution of learned intrinsic attractiveness *μ* of items in two datasets. It shows that most values of *μ* lie around 5 in the APS dataset and 7.5 in the WEIBO dataset respectively, indicating that the average intrinsic attractiveness of messages on micro-blogging network is higher than that of papers in citation network. **(b)** Distribution of learned power-law exponent *γ* in the temporal relaxation function in two datasets. We find that most values of *γ* lie around 1.30 in the APS dataset and shift to around 2.15 in the WEIBO dataset, indicating that the average decaying speed of the attractiveness of messages in micro-blogging network is slower than that of papers in citation network.

Figure 5a illustrates the distribution of intrinsic attractiveness parameter *μ* of items in two datasets. We observe that most values of *μ* lie around 5 in the APS dataset and 7.5 in the WEIBO dataset respectively, indicating that the average intrinsic attractiveness of messages on micro-blogging network is higher than that of papers in citation network. Moreover, for the exponent of power-law temporal relaxation function, parameter *γ*, as shown in Fig. 5b, most values of *γ* lie around 1.30 in the APS dataset. Nevertheless, most values of *γ* shift to around 2.15 in the WEIBO dataset. Note that a smaller *γ* indicates a slower decaying speed of the attractiveness of items. This means that the average decaying speed of the attractiveness of messages in micro-blogging network is slower than that of papers in citation network. One possible explanation for these findings is that micro-blogging system, a typical type of social media for sharing and spreading information, can help messages improve their visibility and prolong their lifespan through a variety of features^38, 39^.

## Discussion

In this report, we propose a general framework to model and predict the dynamic process of collective attention. Our main contribution are three-folds: (1) We proposed a generative probabilistic framework and employed a self-excited Hawkes process to captures the triggering effect of each attention, distinguishing itself from the existing deterministic approaches; (2) We investigated three key ingredients for the dynamics of collective attention and combined them into the proposed model: the intrinsic attractiveness of an item, a reinforcement mechanism corresponding to the “rich-get-richer” effect, and a power-law temporal relaxation function explaining the aging effect in attracting new attentions; (3) We validated the proposed model by applying it on two population-scale datasets. Experimental results demonstrate that the proposed model consistently outperforms the state-of-the-art methods. We hope that this study will provide us richer understanding of the fundamental mechanism of information diffusion and shed light on the collective attention of online human behavior, paving ways towards better management of online content.

The proposed model is flexible, being able to incorporate exogenous information to improve its accuracy. To show this, we consider the inhomogeneous influence between individual attentions. Note that we employ the PageRank score as the influence of a paper in the APS dataset and the logarithmic of the number of a user’s followers in the followship network to represent its influence in the WEIBO dataset (See Supplementary Section S4). We find that when we incorporate the inhomogeneous influence between individuals, the accuracy increases. Therefore, if exogenous information is available, our method can absorb that, improving its predictive power.

There are still a few limitations on the proposed method. Although the overall performance is very well, it does not hold for some abnormal dynamic processes with specific patterns (by using machines, zombie followers, etc.). In addition, maximum likelihood parameter estimation suffers from the over-fitting problem for small sample size. Both of these are very interesting and we will try to solve them in our future work.

A long list of extensions can be conducted based on our findings. Examples include thorough investigation of the effect of the choice of temporal relaxation function, deep exploration on the interplay between the dynamics of collective attention and the structural characteristics of the networks spanned by early adopters, i.e., the users who view or forward the item in the early stage of dissemination. Moreover, it is also an interesting research topic to analyze the effect of the inhomogeneous influence among individuals. In addition, one is also encouraged to enrich the proposed model by incorporating more factors such as, different network behavior for particular types of content extracted from the item itself. More broadly, one is also encouraged to investigate the potential connection between the theoretical approach applied in this paper and the revolution occurring in physics with an increasing interest for renewal processes and ergodicity breaking^40–42^.

## Electronic supplementary material

Supplementary Information
