# Supplementary material for: Uncovering and Predicting the Dynamic Process of Collective Attention with Survival Theory
Source: Sci Rep. 2017 Jun 1;7:2621. doi: 10.1038/s41598-017-02826-6 (PMC5453944; doi:10.1038/s41598-017-02826-6)
Supplement: Supplementary file 1 — Supplementary Information [file 41598_2017_2826_MOESM1_ESM.pdf]

Supplementary Information
Uncovering and Predicting the Dynamic Process of Collective Attention with Survival Theory

Peng Bao[1] Xiaoxia Zhang[2]

1 School of Software Engineering, Beijing Jiaotong University, Beijing, China
2 School of Economics and Management, Tsinghua University, Beijing, China
∗ Corresponding Author: baopeng@bjtu.edu.cn

Contents

1 Datasets **1**

2 Maximum likelihood estimation for model parameters **2**

3 Comparative models **3**

4 Incorporating exogenous information **3**

1 Datasets

The APS dataset used in this report comprises the papers published in all the journals in American Physical Society (APS) from 1893 to 2009, consisting of 245,365 authors, 463,344 papers, and 4,692,026 citations (Table 1). For each paper, the dataset includes title, DOI, author name, institute, printed time, received time, references, PACS code and so on[1]. Basic statistics of the APS dataset is reported in Table 1.

Table 1: Statistics of the APS dataset.

Statistics	Value
Num of papers	463,344
Num of authors	245,365
Num of citations	4,692,026

The WEIBO dataset is a benchmark dataset that was released as a task of the 13th International Conference on Web Information System Engineering

[1] http://journals.aps.org/datasets

(WISE 2012 Challenge)[2]. The dataset contains crawled users and forwarding behaviors between Aug 24, 2009 and Dec 31, 2011 from a Chinese social media website Sina Weibo[3]. In this report, we select messages that was originally posted to Sina Weibo between July 1, 2011 and July 31, 2011. We cleaned the data by removing inactive users and unpopular messages. We also removed spam users who abnormally forward a single message for hundreds of times. To alleviate the effect from activity pattern of users, we only consider the messages posted between 10am and 10pm per day, which is the active period in Sina Weibo system. There are 2.6 million messages. For each message, we collect its forwarding information between July 1, 2011 and August 31, 2011. Basic statistics of the WEIBO dataset is reported in Table 2.

Table 2: Statistics of the WEIBO dataset.

Statistics	Value
Num of users	43,378,576
Num of followships	198,347,101
Num of cascades	2,636,198

2 Maximum likelihood estimation for model parameters

Given the log-likelihood for the dynamics $\{t_k\}$ up to T as

$$\ell = \frac{1}{1-\gamma}X - \mu T \tag{1}$$

where $X = \sum_{i=1}^{N}\Big((1-\gamma)\ln\Big(\mu + \sum_{t_j<t_i}(t_i-t_j)^{-\gamma}\Big) - (T-t_i)^{1-\gamma}\Big)$.

For parameter μ, γ, the optimal values can be found by maximizing the log-likelihood ℓ in equation (1) using the gradient ascent method. The gradients for each parameter are

$$\frac{\partial \ell}{\partial \mu} = \sum_{i=1}^{N}\frac{1}{\mu + \sum_{t_j<t_i}(t_i-t_j)^{-\gamma}} - T$$
$$\frac{\partial \ell}{\partial \gamma} = \frac{1}{1-\gamma}\frac{\partial X}{\partial \gamma} + \frac{X}{(1-\gamma)^2},$$

[2]http://www.wise2012.cs.ucy.ac.cy/challenge.html
[3]http://www.weibo.com

where

$$\frac{\partial X}{\partial \gamma} = \sum_{i=1}^{N} \left(\frac{(\gamma - 1) \sum_{t_j < t_i} (t_i - t_j)^{-\gamma} \ln(t_i - t_j)}{\mu + \sum_{t_j < t_i} (t_i - t_j)^{-\gamma}} - \ln \left(\mu + \sum_{t_j < t_i} (t_i - t_j)^{-\gamma} \right) \right) + \sum_{i=1}^{N} (T - t_i)^{1-\gamma} \ln(T - t_i).$$

According to the standard gradient ascent method, update rules at the n-th iteration are shown as follows.

$$\mu^{(n+1)} = \mu^{(n)} + \eta_1 \cdot \frac{\partial \ell}{\partial \mu}$$
$$\gamma^{(n+1)} = \gamma^{(n)} + \eta_2 \cdot \frac{\partial \ell}{\partial \gamma}$$

where η_1 and η_2 are the learning rate at each iteration. The algorithm stops when the change in an iteration is small enough.

3 Comparative models

Following two baseline models are implemented for comparison.

- The **WSB** model proposed in [1]. Wang et al. employed reinforced Poisson processes, modeling three phenomena: fitness of an item, a log-normal temporal relaxation function and a reinforcement mechanism.
- The **SEISMIC** model proposed in [2]. Zhao et al. employed a double stochastic process, one accounting for infectiousness and the other one for the arrival time of events. It is the current state of the art in predicting dynamics of popularity.

4 Incorporating exogenous information

The proposed model is flexible, being able to incorporate exogenous information such as structure features, to improve its accuracy. To show this, we consider the inhomogeneous influence between individuals. Hence the rate function is modified as follows

$$\lambda(t) = \mu + \sum_{0 < t_i < t} a_i \ \phi(t - t_i), \tag{2}$$

where μ is the intrinsic attractiveness of the item, $\phi(\tau)$ is the relaxation function that characterizes the temporal inhomogeneity due to the aging effect, a_j is the triggering strength of each subsequent attention, capturing the influence of individuals. Note that we employ the page rank score as the influence of a paper in the APS dataset and the logarithmic of the number of a user's followers in the followship network to represent its influence in the WEIBO dataset.

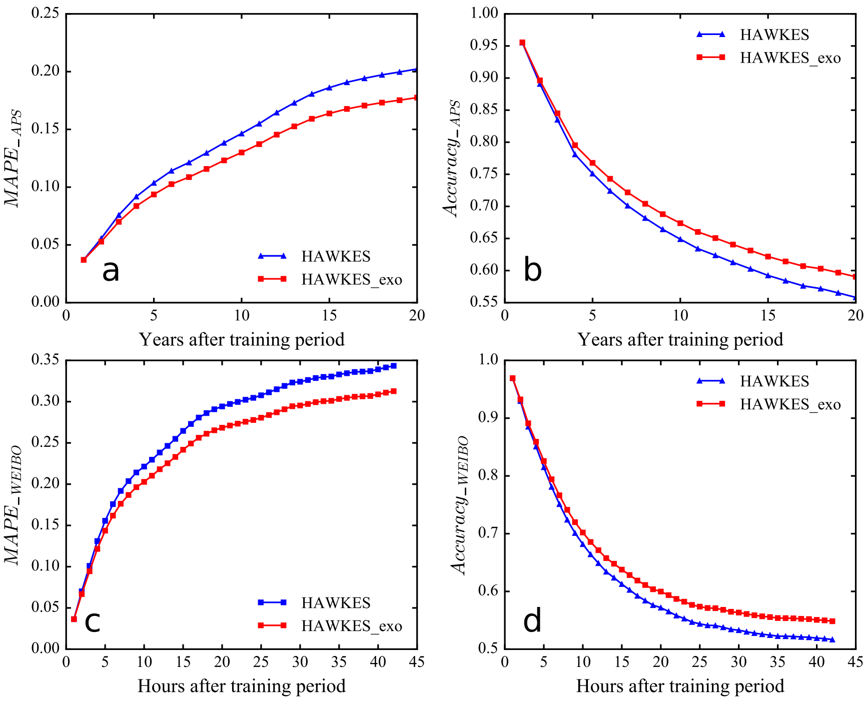

Figure 1: **Prediction results.** **(a)** $MAPE$ of model prediction with respect to the 1st to 20th year after the training period in the APS dataset. **(b)** *Accuracy* of model prediction with respect to the 1st to 20th year after the training period in the APS dataset. **(c)** $MAPE$ of model prediction with respect to the 1st to 42nd hour after the training period in the WEIBO dataset. **(d)** *Accuracy* of model prediction with respect to the 1st to 42nd hour after the training period in the WEIBO dataset.

We find that when we incorporate the inhomogeneous influence between individuals, the accuracy increases. Therefore, if exogenous information is available, our method can absorb that, improving its predictive power.

References

[1] Wang, D., Song, C. & Barabási, A.-L. Quantifying long-term scientific impact. *Science* **342,** 127–132 (2013).

[2] Zhao, Q., Erdogdu, M. A., He, H. Y., Rajaraman, A. & Leskovec, J. SEISMIC: a self-exciting point process model for predicting tweet popularity. *Proc. SIGKDD '15*, 1513–1522 (2015).
